# Preparation of a Functional Rat LDL Receptor Minigene

**Published:** 2019-12-31

**Authors:** Catherine J. Wooten, Dayami Lopez

**Affiliations:** Department of Pharmaceutical Sciences, Biomanufacturing Research Institute and Technology Enterprise (BRITE), College of Arts and Sciences, North Carolina Central University, Durham, USA

**Keywords:** LDL receptor, Minigene, Cloning, Research tool, Transcriptional regulation

## Abstract

The majority of the low-density lipoprotein (LDL) receptors present in the body are expressed in the liver. Therefore, plasma LDL levels significantly correlate with changes in the activity of the hepatic LDL receptor. Based on this, there is a need to understand the regulatory mechanisms that control the hepatic expression of the low-density lipoprotein (LDL) receptor. Herein, we have prepared a functional rat LDL receptor minigene construct that can produce mRNA after splicing. Sequence analysis suggests that this construct has the potential to code for a truncated version of LDL receptor protein. This minigene could be used as a research tool to identify small molecules, natural products, and regulators of the LDL receptor gene that could be developed into LDL receptor-specific activators for therapeutic use.

## Introduction

The primary determinant of plasma low density lipoprotein (LDL) levels is the hepatic LDL receptor [1–3]. The liver expresses about 70% of the total LDL receptors present in the body so that plasma LDL levels usually vary in correlation with changes in the activity of the hepatic LDL receptor [3]. Mutations in the LDL receptor gene have been identified as the main cause of familial hypercholesterolemia (FH1; MIN # 606945) [4–7]. Heterozygous FH patients usually have low hepatic uptake of LDL, increased plasma LDL concentrations, and premature cardiovascular disease [7]. Although rare to find, homozygous FH patients have extremely low or absent plasma clearance of LDL, substantially raised LDL concentrations, and accelerated development of cardiovascular disease [7].

The 45 kb-long human LDL receptor gene is located on chromosome 19p13.1–13.3 [8]. The promoter of this gene has two TATA-like sequences and three 16-bp direct repeats critical for gene transcription [9]. Repeats 1 and 3 are recognized by the transcription factor Sp1 and are essential for basal transcription levels of the LDL receptor, in the presence or absence of sterols [9, 10]. Repeat 2 contains a sterol regulatory element (SRE) that controls transcription of the LDL receptor in response to cholesterol levels [11]. Other critical transcriptional response elements identified in the promoter of the LDL receptor gene are thyroid hormone response elements, which are responsible for the hypercholesterolemia typically seen in hypothyroid patients [12, 13]. There is about 78% similarity between the human, mouse, and rat LDL receptor genes (http://www.genecards.org/cgi-bin/carddisp.pl?gene-LDLR).

The LDL receptor is a mature type 1 transmembrane protein containing 839 amino acids and five functionally distinct domains [14]. The ligand binding domain (determined by exons 2 to 6) is at the N-terminal region and is composed of seven adjacent LDL receptor type-A (LA) repeats [15]. Each LA repeat uses three conserved calcium-binding acidic residues for protein-protein interactions [16]. The second domain is the epidermal growth factor (EGF) precursor domain (determined by exons 7 to 14) [15]. This domain is composed of two EGF repeats (EGF-A and EGF-B), the YWTD region, and a third EGF repeat (EGF-C) [15]. The YWTD region folds into a six-bladed μ-propeller, which is involved in the release of lipoprotein particles from the receptor within the endosome [16, 17]. Next is the O-linked glycosylation domain (encoded by exon 15), which is rich in serine and threonine residues that get glycosylated in the Golgi leading to an increase in the molecular weight of the newly synthesized receptor from 120 kDa up to 160 kDa [15, 18]. The following region is the transmembrane domain (encoded by exons 16 & 17) that anchors the receptor to the plasma membrane [15]. The final domain is the 50-residues cytoplasmic tail (encoded by exons 17 &18), which is necessary for receptor localization in clathrin-coated pits and endocytosis [19].

There is a need to understand the regulatory mechanisms that control the hepatic expression of the LDL receptor including identification of transcriptional regulators of this gene. Herein, we have prepared a rat LDL receptor minigene construct that could assist in the identification of transcriptional activators of the LDL receptor with potential therapeutic use.

## Materials and Methods

### Materials

The whole rat genomic DNA used as template in PCR reactions was from BD Biosciences (San Jose, CA). Primers and oligonucleotides were synthesized by Eurofins Genomics (Huntsville, AL). Invitrogen ThermoFisher Scientific (Carlsbad, CA) was the source of Taq DNA polymerase, dNTPs (blended deoxynucleotide triphosphates), the pCR 2.1 TA cloning vector, the One Shot® INVF’ Chemically Competent bacterial cells, the T4 DNA ligase kit, 2 × PCR Master Mix, low glucose (5.55 mM) Dulbecco’s modified Eagle’s medium (LG-DMEM), standard fetal bovine serum (FBS), antibiotics (penicillin/streptomycin solution), the AB reverse transcriptase system, and AB SYBR Green PCR Master Mix were from Invitrogen ThermoFisher Scientific (Carlsbad, CA). The 1-kb and 100-bp DNA ladders, all the restriction enzymes, the FuGENE® 6 Transfection Reagent, and the DNA removal kit were purchased from Promega (Madison, WI). The GenElute extraction kit was from Sigma-Aldrich (St. Louis, MO). The Qiagen plasmid prep kit was obtained from Qiagen (Germantown, MD). The original (unmodified) pSG5 vector was purchased from Stratagene (La Jolla, CA). The QuickChange Site-Directed Mutagenesis Kit was obtained from Agilent (Santa Clara, CA). American Type Culture Collection (Manassas, VA) supplied the human hepatocyte-like C3A cell line. The Molecular Research Center (Cincinnati, OH) was the source of TRI Reagent. Other chemicals not mentioned in this section were from Fisher Scientific (Pittsburgh, PA).

### PCR Amplification of the Minigene Fragments

**Figure 1:**
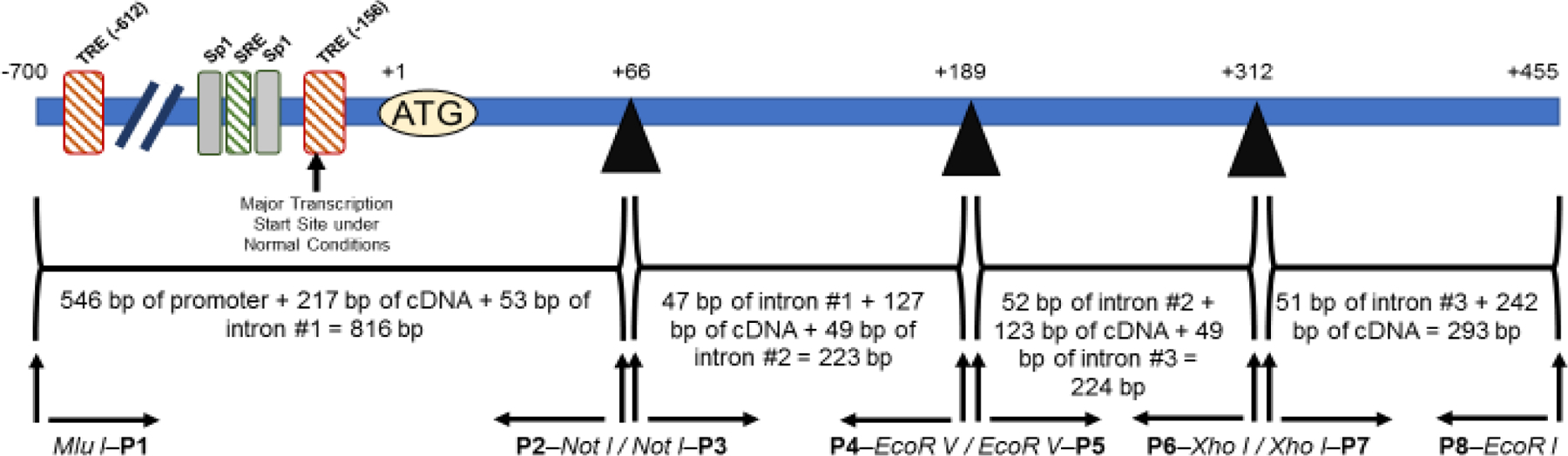
Schematic representation of the assembled rat LDL receptor minigene. This LDL receptor minigene contained sections of the promoter, exons 1–3, partial sequences of exon 4, and sufficient intronic (1–3) sequence to allow splicing of the minigene. Essential motifs found in the promoter of the minigene are illustrated. The position of the eight primers P1–P8 used to clone these four main regions of the minigenes, as well as the restriction enzyme sites added to each primer to facilitate assembling, are also shown. After assembling, the size of the minigene, including restriction enzyme sites, should be 1.588 kb.

**Figure 2:**
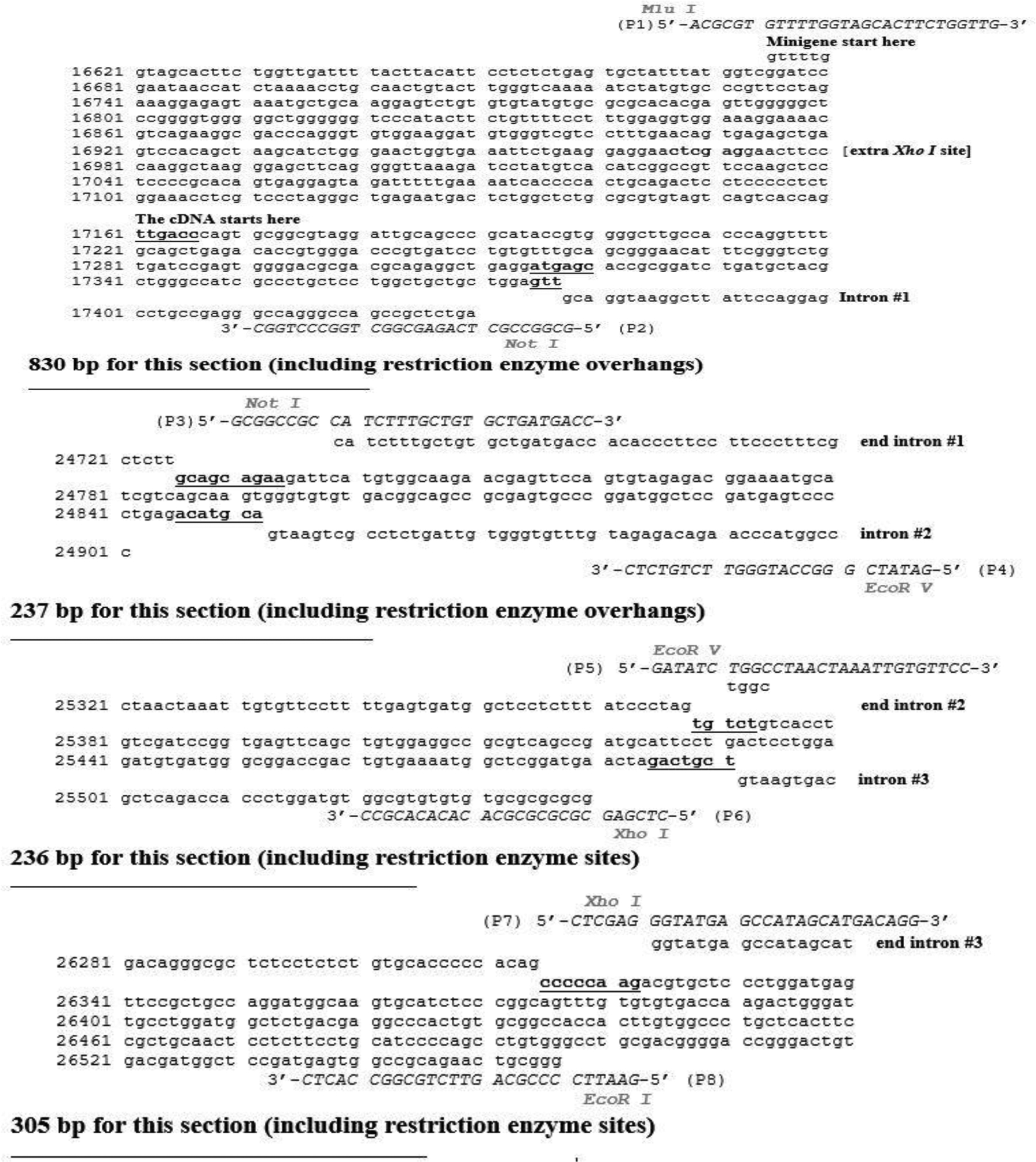
The sequence of the rat LDL receptor minigene confirmed using sequencing. Primers used for cloning are aligned to the sequence. The regions corresponding to each part of the gene have been labeled. The sizes of each minigene fragment are shown.

PCR reactions to amplify the individual genomic fragments for the rat LDL receptor minigene were set as follows: 10 μL of whole rat genomic DNA (stock concentration=0.1 μg/μL), 0.5 μl of Taq DNA polymerase (stock concentration = 1 units/μL), 5 μL of 10 × Buffer, 1 μL of dNTPs (stock concentration = 100 mM), 2 μL of primer mix (stock concentration = 0.1 μg/μL), and 31.5 μL of nuclease-free water. The total volume of each PCR reaction was 50 μL. Each primer mix was designed to be specific for a genomic fragment. To clone genomic fragment #1 (830 bp), primers P1 and P2 were mixed. To clone genomic fragment #2 (237 bp), primers P3 and P4 were mixed. To clone genomic fragment #3 (236 bp), primers P5 and P6 were mixed. To clone genomic fragment #4 (305 bp), primers P7 and P8 were mixed. These primers (P1–P8) were designed to include restriction enzymes at their 5’-end to facilitate their assembling into the minigene. Figure 1 shows the positions of these primers in each section of the minigene. The primer sequences (also shown in Figure 2) were: P1: 5’-ACGCGT GTTTTGGTAGCACTTCTGGTTG-3’; P2: 5’-GCGGCCGC TCAGAGCGGCTGGCCCTGGC-3’; P3: 5’-GCGGCCGC CATCTTTGCTGTGCTGATGACC-3’; P4: 5’-GATATC GGGCCATGGGTTCTGTCTC-3’; P5: 5’-GATATC TGGCCTAACTAAATTGTGTTCC-3’; P6: 5’-CTCGAG CGCGCGCGCACACACACGCC-3’; P7: 5’-CTCGAG GGTATGAGCCATAGCATGACAGG-3’; and P8: 5’-GAATTC CCCGACGTTCTGCGGCCACTC-3’. The restriction enzyme sites (shown in bold, italic letters) were Mlu I in P1; Not I in P2 and P3; EcoR V in P4 and 5; Xho I in P6 and P7; and EcoR I in P8.

PCR amplifications were done using an Eppendorf thermal cycler with the following parameters: 1 cycle at 95 °C for 1 minute, followed by 35 cycles of denaturation at 95 °C for 1 min, annealing at 60 °C for 1 min, and extension at 72 °C for 2 min. To confirm the presence of a single DNA fragment in each PCR reaction, DNA electrophoresis was performed using a 1% agarose gel in 1 × TAE buffer/0.5 μg/mL of ethidium bromide. Electrophoresed DNA samples were visualized using the Kodak Image Station 4000R Pro Imaging System and the Kodak Molecular Imaging software (New Haven, CT). The sizes of the amplified DNA fragments were estimated by comparing to a 100-bp DNA ladder. PCR products of the correct size were eluted from the DNA gel using the GenElute extraction kit and cloned into the pCR 2.1 TA cloning vector according to the manufacturers’ protocols. After transformation into One Shot® INVF’ Chemically Competent cells, growing, and isolation using a Qiagen plasmid prep kit, the plasmids were sequenced by Eurofins Genomics (Louisville, KY) to confirm the presence of the expected genomic fragments. Plasmids containing the expected genomic regions were used in follow-up studies.

### Site-Directed Mutagenesis

This technique was used to prepare the modified (m) pSG5 (mpSG5) vector and the mutated versions of the rat LDL receptor minigene-mpSG5. The protocol was performed as previously described [13]. Oligonucleotides used to insert the Mlu I site into the pSG5 vector, so the site can be used to clone in the assembled rat LDL receptor minigene, were 5′-GAACCAGCTGTGGAACGCGTGTCAGTTAGGGTGTGG-3′ and its complement. Oligonucleotides used to mutate the LDL receptor promoter within the assembled-minigene were 5′-CTGCGCGTGTGATCAGTCACCGTCCTGCCCAGCGCGGCGTAG-3′ and its complement for the motif at −156 (−156 mutant), and 5′-CCTGCAACTGTACTTAAATTAAAAATCTATGTGCCCG-3′ and its complement for the motif at −612 (−612 mutant). The nucleotides that are underlined Correspond to the modified/mutated bases that were introduced. After PCR amplification, the original DNA templates were digested with Dpn I according to the protocol, whereas the modified/mutated newly synthesized DNAs were transformed into competent cells according to the manufacturer’s instructions. Plasmid preparation was done using the Qiagen protocol as described above. The modification in the mpSG5 vector was confirmed using restriction enzyme analysis. Mutations in the rat LDL receptor minigene were confirmed by sequencing also described above.

### Assembling of the Rat LDL Receptor Minigene

Restriction enzyme reactions were performed using 1 μL of DNA sample (stock concentrations = 0.1–1 μg/μL), 19 μL of nuclease-free water, 2.5 μL of 10 × Buffer, and 2.5 μL of enzyme mixture (one or two enzymes, depending on the reaction). Reactions were incubated at 37 °C for 30 minutes to allow digestion of the DNA samples. The resulting digested products were analyzed by DNA electrophoresis as described in the previous section. After imaging, inserts and vectors required for ligation were eluted from the DNA gels using the GenElute extraction kit. Ligations reactions were performed overnight using T4 DNA ligase.The pCR 2.1 vector containing fragment #3 was digested with Xho I/Xba I to linearize the vector. The vector containing fragment #4 was digested with Xho I/Spe I to cut out the insert (fragment #4). The linearized vector containing fragment #3 and the fragment #4 insert were isolated from the gel and then ligated. Ligation reactions were transformed into OneShot competent cells as described above. Colonies were grown, and plasmids isolated and tested for the inclusion of the two fragments using restriction enzyme analysis. The resulting vector containing fragments #3–#4 and the plasmid containing fragment 2 were digested with EcoR V/Spe I to linearize the vector and cut out the insert (fragment #2), respectively. The linearized vector containing fragments #3–#4 and the fragment #2 insert were isolated from the gel and ligated using T4 DNA ligase. Again, after transformation into competent bacterial cells, colonies were grown, and plasmids were isolated and tested for the inclusion of the three genomic fragments as described earlier. The resulting vector containing fragments #2-#3-#4 and the plasmid containing fragment #1 were digested with Not I/Spe I to linearize the vector and cut out the insert (fragment #1), respectively. The linearized vector containing fragments #2-# 3-#4 and the fragment #1 insert were once again isolated from the gel and ligated using T4 DNA ligase. After transformation into competent bacterial cells, colonies were grown, and plasmids were prepared and tested for the inclusion of all four fragments. The resulting vector contained the assembled-minigene, which was next inserted into the mpSG5 vector. For this part, the assembled-minigene-pCR 2.1 plasmid and the mpSG5 vector prepared by site-directed mutagenesis were digested with Mlu I/EcoR I to cut out the assembled-minigene insert and the mpSG5 vector region to be replaced by the minigene. The digested mpSG5 vector and the assembled-minigene insert were isolated from the gel and ligated using T4 DNA ligase. Colonies obtained after transformation into OneShot competent bacterial cells were grown and tested for the inclusion of the assembled-minigene using different restriction enzymes. The resulting rat LDL receptor minigene-mpSG5 construct was used in various tests, including restriction enzyme analysis, and in site-directed mutagenesis to prepare minigene mutants as described above.

### Testing of the Minigene Constructs using Standard PCR

PCR reactions used to test the amplification of sections of the assembled-minigene-mpSG5 construct were as follow: 5 μL of template (minigene-mpSG5 constructs or empty mpSG5 plasmid; stock concentration = 2 ng/μL), 25 μl of 2 × PCR Master Mix, 2 μL each of each primer (stock concentration = 0.05 μg/μL), and 16 μL of nuclease-free water. The empty mpSG5 vector (negative control) and the minigene-mpSG5 construct were tested in reactions with the sequencing primer (1) and antisense primer(3). The minigene-mpSG5 construct was also tested in a reaction with the sense primer (2) and antisense primer (3). The minigene-mpSG5 construct along with three mutants were tested in reactions including sequencing primer (1) and new antisense primer (5). The sequences of these primers were 5’-GCGATTAAGTTGGGTAACGC-3’ for sequencing primer (1); 5’-TGAGCACCGCGGATCTGATG-3’ for sense primer (2); 5’-GTAACCATTATAAGCTGC-3’ for antisense primer (3); and 5’-ACCATTATAAGCTGC-3’ for new antisense primer (5). The sequencing primer (1) and antisense primer (3) were specific for the mpSG5 regions around the assembled minigene. The sense primer (2) was specific for the rat LDL receptor cDNA sequence within fragment #1.The new antisense primer (5) was designed to the region of the mpSG5 vector that was transcribed as part of the minigene mRNA sequence. These primers were different from the eight primers used to amplify the LDL receptor genomic regions employed in the assembling of the minigene described above. PCR amplifications were done using an Eppendorf thermal cycler with the following parameters: 1 cycle at 95 °C for 2 minutes, followed by 40 cycles of denaturation at 95 °C for 1 min, annealing at 55 °C for 1 min, and extension at 72 °C for 2 min. To confirm the presence of a single DNA fragment after amplification, DNA electrophoresis was performed. The sizes of the amplified fragments were estimated by comparing to the DNA ladder.

### Transfections into Human Hepatocyte-like C3A Cells

Cells were cultured in LG-DMEM medium supplemented with 10% FBS and antibiotics, at 37°C, with humidified atmosphere and 5% CO. For 2 experiments, cells were plated in 12-well plates, and 24 h later, they were transfected with the rat LDL receptor minigene-mpSG5 constructs, 1 μg DNA per well, using the FuGENE® 6 transfection reagent protocol [12]. Briefly, each plasmid to be transfected was incubated with FuGENE®6 (3:1; transfection reagent: DNA) in 100 μL of medium for 20 min. The DNA–FuGENE®6 complexes were then added to the cells containing fresh medium.Cells were incubated for 48 h at 37°C before they were used in the preparation of RNA.

### RNA Preparation and Quantitative Real-time PCR (qRT-PCR)

The isolation of RNA samples was performed using the TRI reagent method as previously described [20]. The concentrations and purity of the RNA samples were determined using a Nanodrop 2000. RNA electrophoresis also confirmed the integrity of the RNA. DNase I treatment and reverse transcriptase reactions were carried out using standard methods. The qRT-PCR reactions were performed with 100 ng of ssDNA, the Applied Biosystems SYBR Green PCR Master Mix, and the AB real-time PCR system, with the following parameters: denaturation at 95°C for 10 minutes, and then 45 cycles of denaturation at 95°C for 30 seconds, annealing at 60°C for 15 seconds, extension at 72°C for 30 seconds, and photo documentation at 80°C for 15 seconds. A melt curve was proceeded to determine if each primer set was amplifying as a single band. The minigene-specific primers for qRT-PCR were new sense primer (4) and new antisense primer (5). The new sense primer (4) was designed to overlap the junction between exons 3 and 4 within the rat LDL receptor minigene. The sequence for the new sense primer (4) was 5’-CTAGACTGCTCCCCCAAGAC-3’. The new sense primer (4) could be used in qRT-PCR only since splicing of the minigene was required before it could anneal to the sequence. The new antisense primer (5) was designed as described in the standard PCR section and could be used in both standard and qRT-PCR. The primers 5’-GGGACAAGTGGCGTTCAG-3’ and 5’-CGCTGAGCCAGTCAGTGTAG-3’ to detect human 18s rRNA were used as internal control for the RNA preparation and the calculations. The sizes of the amplified PCR fragments were 300 bp for the minigene and 100 bp for 18s rRNA. The Comparative CT method was used for quantitation as previously reported [20]. Data from at least three independent measurements (n = 3) per construct were compared employing analysis of variance (ANOVA)followed by Dunnett’s multiple comparison tests. The significance level was set at α = 0.05. All the calculations and graphing were done using the GraphPad Prism 7 software (GraphPad Software, Inc., La Jolla, CA). Some aliquots of the qRT-PCR reactions were also analyzed using DNA electrophoresis, and the sizes of the DNA fragments were estimated by comparing to a 100-bp DNA ladder.

## Results and Discussion

Eight primers (P1–P8; Figure 1 and 2) were designed (containing specific restriction enzymes at the 5’-end of each primer), synthesized, and employed in PCR reactions using whole rat genomic DNA as the template. The genomic sequence used to design the primers was obtained from GenBank (chromosome 8, location 8q13, Gene ID # 300438, Source RGD:2998) and information in a previous publication [13]. Figure 1 shows the positions of the primers P1–P8 in each section of the minigene. Sequences for these primers are shown in Figure 2. The position of the different introns was obtained by aligning the genomic sequence from GenBank with the rat LDL receptor cDNA sequence [21]. About 50 bp on each end of the intronic regions was cloned since it has been predicted that all the intronic regulatory regions are located within the first 48 nucleotides upstream of the 5’-splice site [22]. PCR reactions were performed as described under Materials and Methods. All four genomic regions (830 bp, 237 bp, 236 bp, and 305 bp; see Figure 2), labeled fragments #1 through #4, respectively, were successfully amplified. Resulting PCR fragments were individually cloned into the pCR2.1 TA cloning vector, and their sequences were confirmed in both directions.

**Figure 3:**
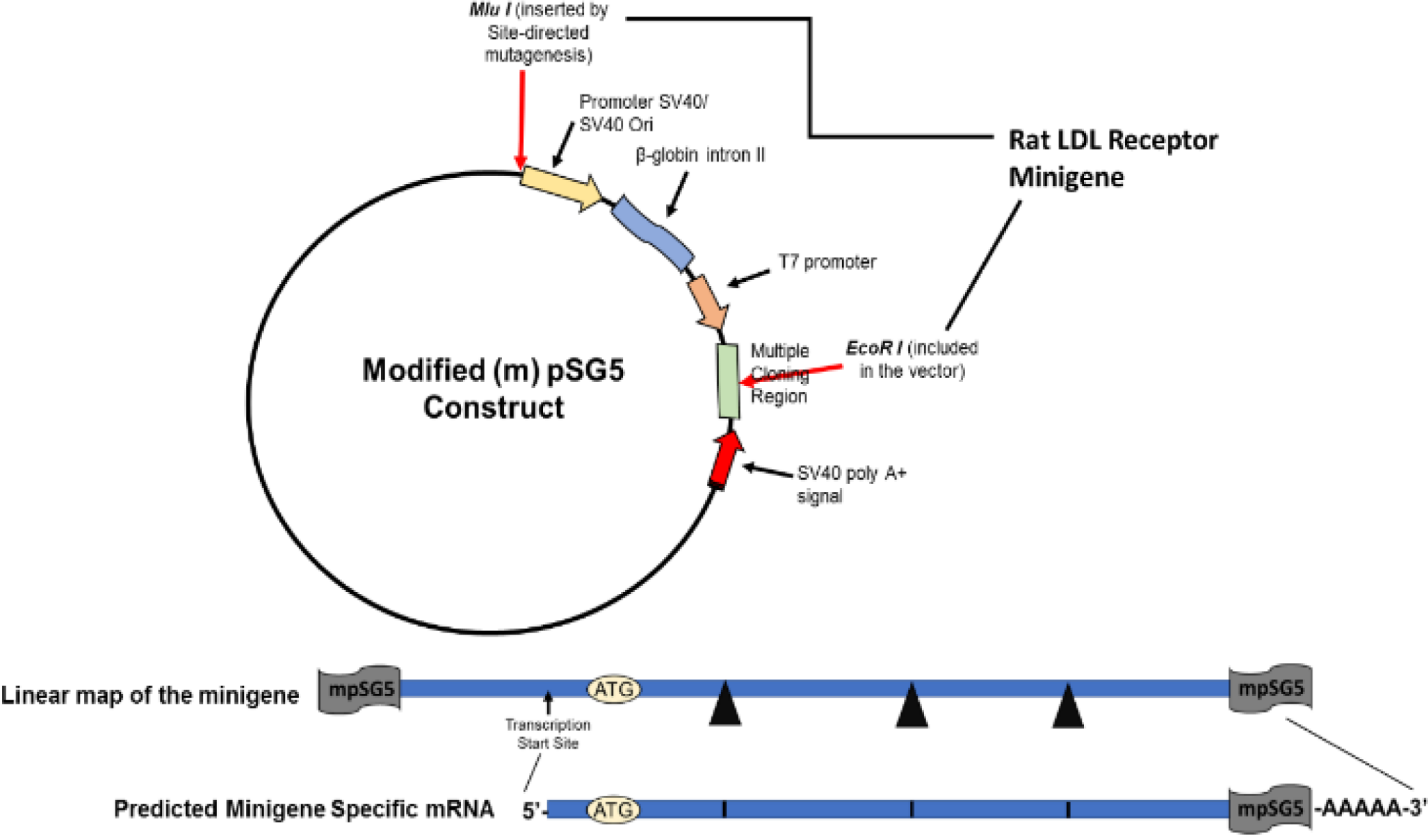
Schematic structure of the modified (m) pSG5 construct indicating which regions of the original vector are replaced with the assembled rat LDL receptor minigene. Schematics of the linear map of the minigene in mpSG5 and the predicted minigene-specific mRNA that should be produced from the construct are also shown.

**Figure 4:**
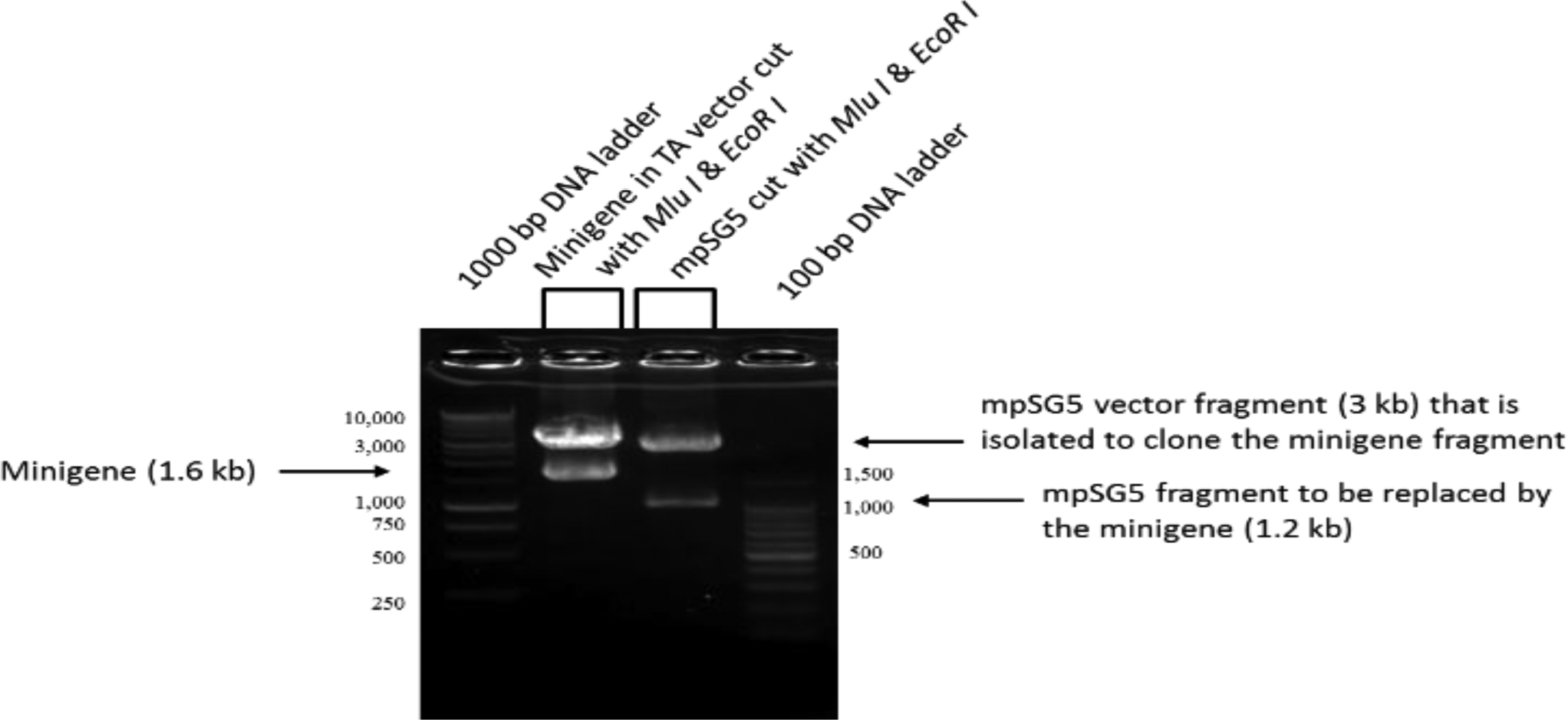
DNA electrophoresis showing the cutting of the assembled LDL receptor minigene-PCR 2.1 plasmid and the mpSG5 vector (empty) with Mlu I/EcoR I. The main DNA fragments obtained after the digestion reactions are indicated using arrows. The sizes of the DNA fragments were estimated by comparing to the 1000-bp and 100-bpDNA ladders.

The minigene was first assembled into the pCR2.1 TA cloning vector using restriction enzymes Mlu I/Not I for the 830 bp fragment; Not I/EcoR V for the 237 bp fragment; EcoR V/Xho I for the 236 bp fragment; and Xho I/EcoR I for the 305 bp fragment. The order in which the fragments were combined to assemble the minigene is described under Materials and Methods. The assembled-minigene was then subcloned into a modified (m) pSG5 (mpSG5) vector using the restriction enzymes Mlu I/EcoR I, which cuts around the assembled minigene in the pCR2.1 TA vector. The original (unmodified) pSG5 vector is a eukaryotic expression vector characterized by having the SV40 early promoter and SV40 polyadenylation signal to promote expression in vivo, the T7 bacteriophage promoter to facilitate in vitro transcription of cloned inserts, and the β-globin intron II to allow splicing of expressed transcripts. Specific details about the assembling of the minigene in pCR2.1 and the modification of the pSG5 vector are included in Materials and Methods. Since our interest was to prepare a minigene that could be used to find transcriptional regulators of the rat LDL receptor without affecting the normal splicing of the minigene, the pSG5 vector region containing the SV40 early promoter, the β-globin intron II, and the T7 bacteriophage promoter were replaced with the assembled rat LDL receptor minigene. Only the SV40 polyadenylation signal remained in the vector after the Restriction enzyme analysis was conducted next. Figure 4 shows the cutting of the LDL receptor minigene-pCR insertion of the assembled minigene. These changes in the mpSG5 vector did not affect the vector’s ability to replicate into bacterial cells (data not shown). To perform this substitution, the original vector was modified to include a Mlu I restriction enzyme site upstream of the SV40 promoter using site-directed mutagenesis. This modification of the pSG5 vector was done before subcloning the assembled minigene into this vector. The insertion of the Mlu I site into the pSG5 vector was confirmed using restriction enzyme analysis. Figure 3 shows the parts of the pSG5 vector (the SV40 early promoter, the β-globin intron II, and the T7 bacteriophage promoter) replaced by the assembled rat LDL receptor minigene. The promoter to control the expression of the rat LDL receptor minigene was provided by the minigene itself. The empty mpSG5 vector, which is the mpSG5 vector before the insertion of the assembled rat LDL receptor minigene, was used as the negative control in the confirmation experiments reported below.

2.1 plasmid and the empty mpSG5 vector using Mlu I/EcoR I.

**Figure 5:**
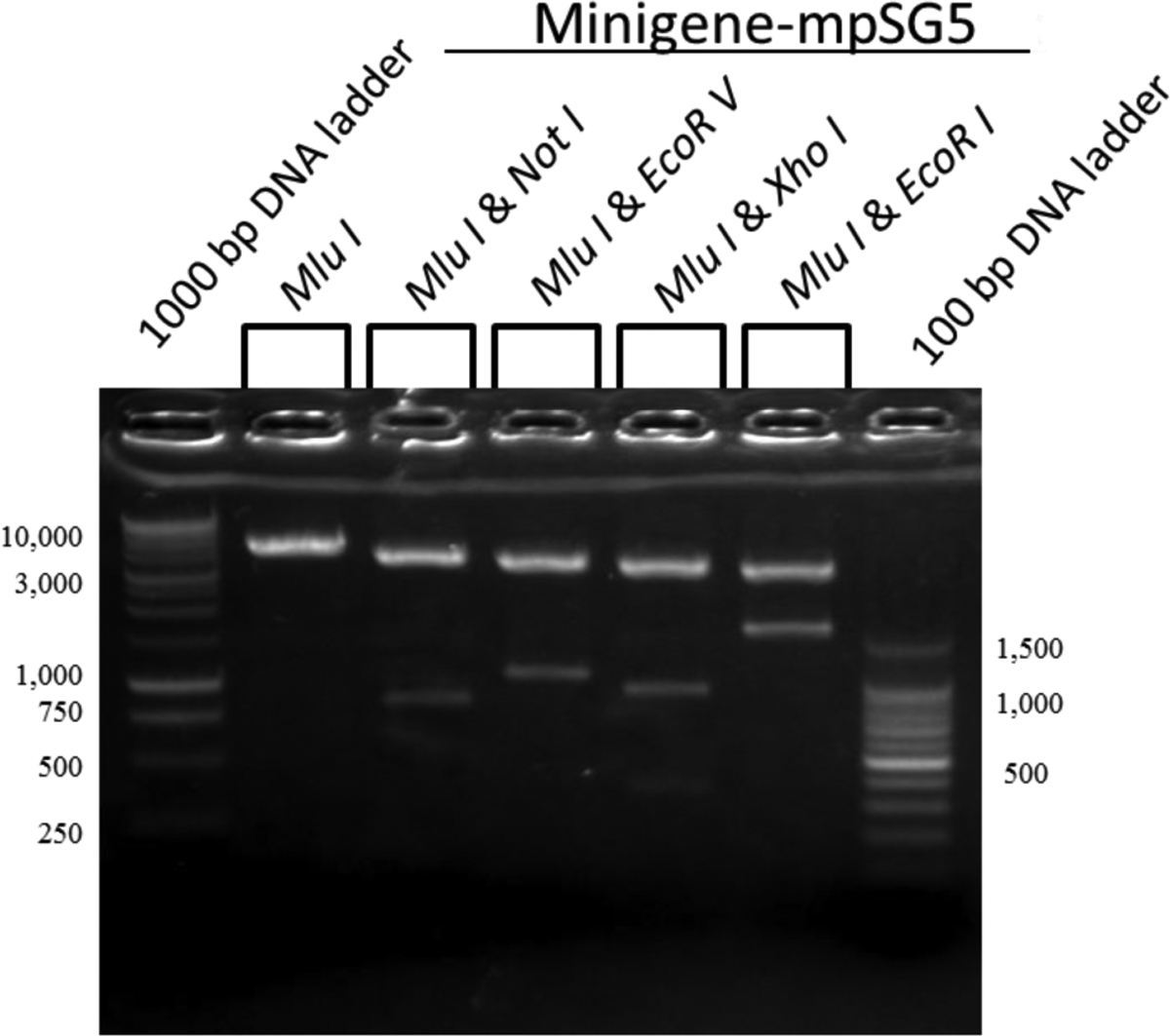
Restriction enzyme map of the assembled-minigene-mpSG5 construct. The construct was digested with different combinations of restriction enzymes to release specific regions of the minigene. The sizes of the resulting DNA fragments after digestion and electrophoresis were determined by comparing to the 1000-bp and 100-bp ladders.

The size of the minigene fragment was about 1.6 kb, whereas the size of the DNA fragment cut out from the mpSG5 vector (later replaced with the minigene) was about 1.2 kb. The 1.6 kb assembled minigene insert was subsequently ligated to mpSG5 vector (about 3 kb) using T4 DNA ligase. The size of the assembled-minigene-mpSG5 construct after cloning was 4.6 kb. Figure 5 shows a restriction enzyme map of the minigene-mpSG5 construct. As shown, digesting with Mlu I alone linearized the plasmid that had a size of 4.6 kb. Digesting with Mlu I/Not I released fragment #1 from the construct resulting in two DNA fragments of 3.75 kb (vector-fragments #2–#4) and 850 bp (fragment #1), respectively. Digesting with Mlu I/EcoR V released fragments #1–#2 from the construct resulting in two DNA fragments of about 3.5 kb (vector-fragments #3–#4) and 1.1 kb (fragments #1-#2), respectively. Digesting with Mlu I/Xho I released fragments #1-#3 from the construct, but it also cut this genomic region at about 400 bp from the 5’-end (see Figure 2 for information about this restriction enzyme site). This resulted in three DNA fragments of about 3.3 kb (vector-fragment #4), 400 bp (corresponding to the beginning of fragment #1), and 920 bp (the remaining of fragment #1-#2-#3), respectively.

**Figure 6:**
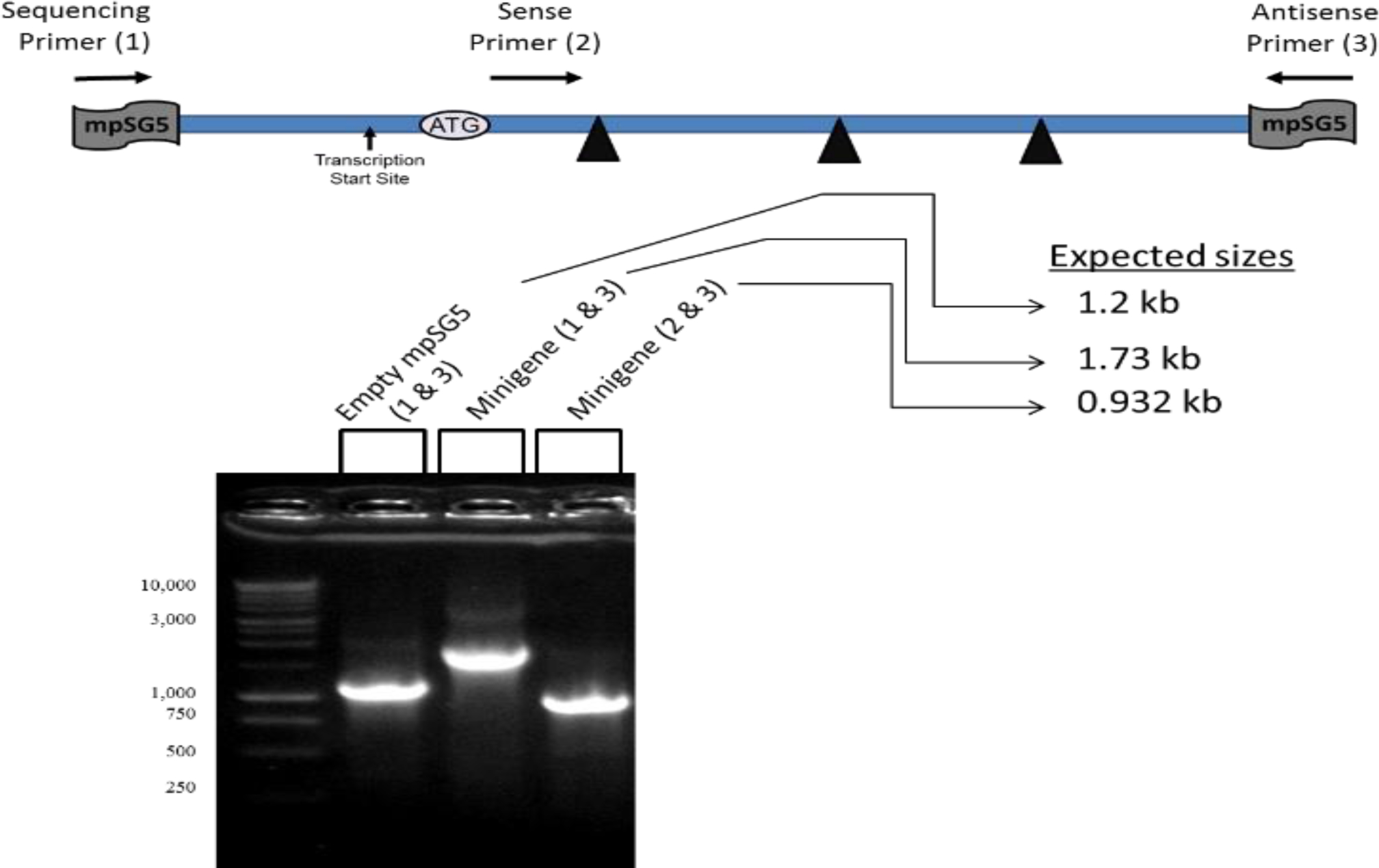
Testing of the minigene using standard PCR. A schematic representation of the position of the primers used in the PCR relative to the minigene map is shown. A typical DNA electrophoresis of the PCR reactions performed with the empty mpSG5 vector and the minigene-mpSG5 construct is illustrated. The primers used in each reaction and the expected sizes of the products are indicated.

Digesting with Mlu I/EcoR I released the assembled minigene from the vector resulting in two DNA fragments of about 3 kb (empty vector) and 1.6 kb (assembled minigene), respectively. The minigene-mpSG5 construct was also tested using a combination of primers and standard PCR. The position of the primers in the minigene-mpSG5 construct is shown in Figure 6. The expected sizes of the DNA regions that were amplified using each primer set, according to the genomic sequences of the mpSG5 vector and the minigene-mpSG5 construct, are also shown in Figure 6. Mutated versions of the rat LDL receptor minigene-mpSG5 construct were also prepared. For this, site-directed mutagenesis was employed as described under Materials and Methods. The mutations inserted have been previously shown to affect the thyroid hormone regulation of the rat LDL receptor gene [12, 13, 23]. The presence of these mutations within the LDL receptor promoter in the minigene was confirmed using sequencing. This process resulted in four minigene-mpSG5 constructs: wild-type (WT, no modifications), -As depicted, using the sequencing primer (1) and antisense primer (3), the expected sizes of 1.2 and 1.73 kb for the empty mpSG5 vector (still containing the region later replaced by the assembled-minigene) and the minigene-mpSG5 construct, respectively, were obtained. Amplifying using the sense primer (2) and antisense primer (3) resulted in a DNA fragment of about 930 bp which was the expected fragment size when using the minigene-mpSG5 construct as the template for the PCR reaction.612 mutant, −156 mutant, and double (Db) mutant (including both −612 and −156 mutations).

**Figure 7:**
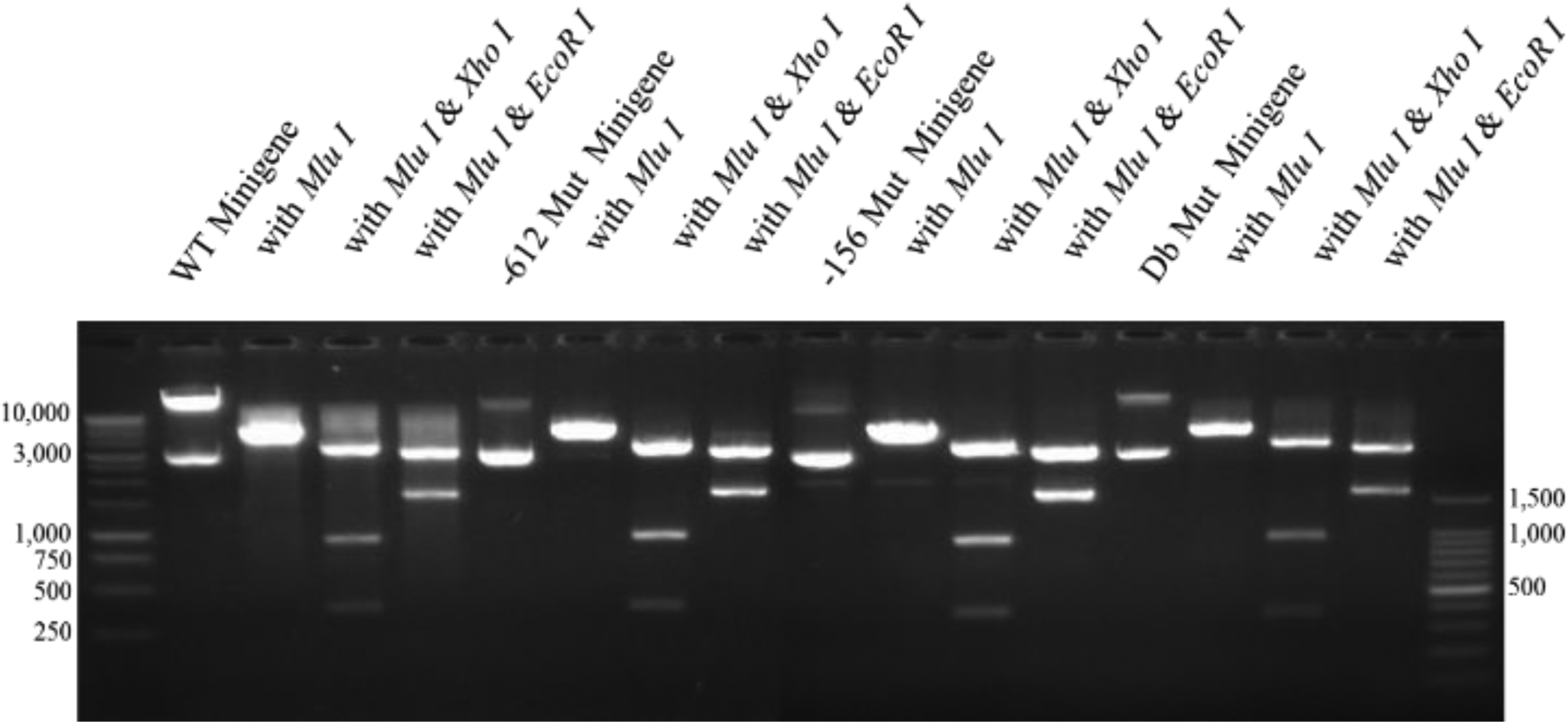
Testing four minigene-mpSG5 constructs using restriction enzyme analysis.The wild-type (WT) and three mutated versions (−612, −156, and Db) of the minigene-mpSG5 were employed in this test. The mutated constructs were prepared using site-directed mutagenesis as indicated under Materials and Methods. Three different restriction enzyme reactions were used to test each construct. The restriction enzymes used are indicated. A typical DNA electrophoresis is shown.

**Figure 8:**
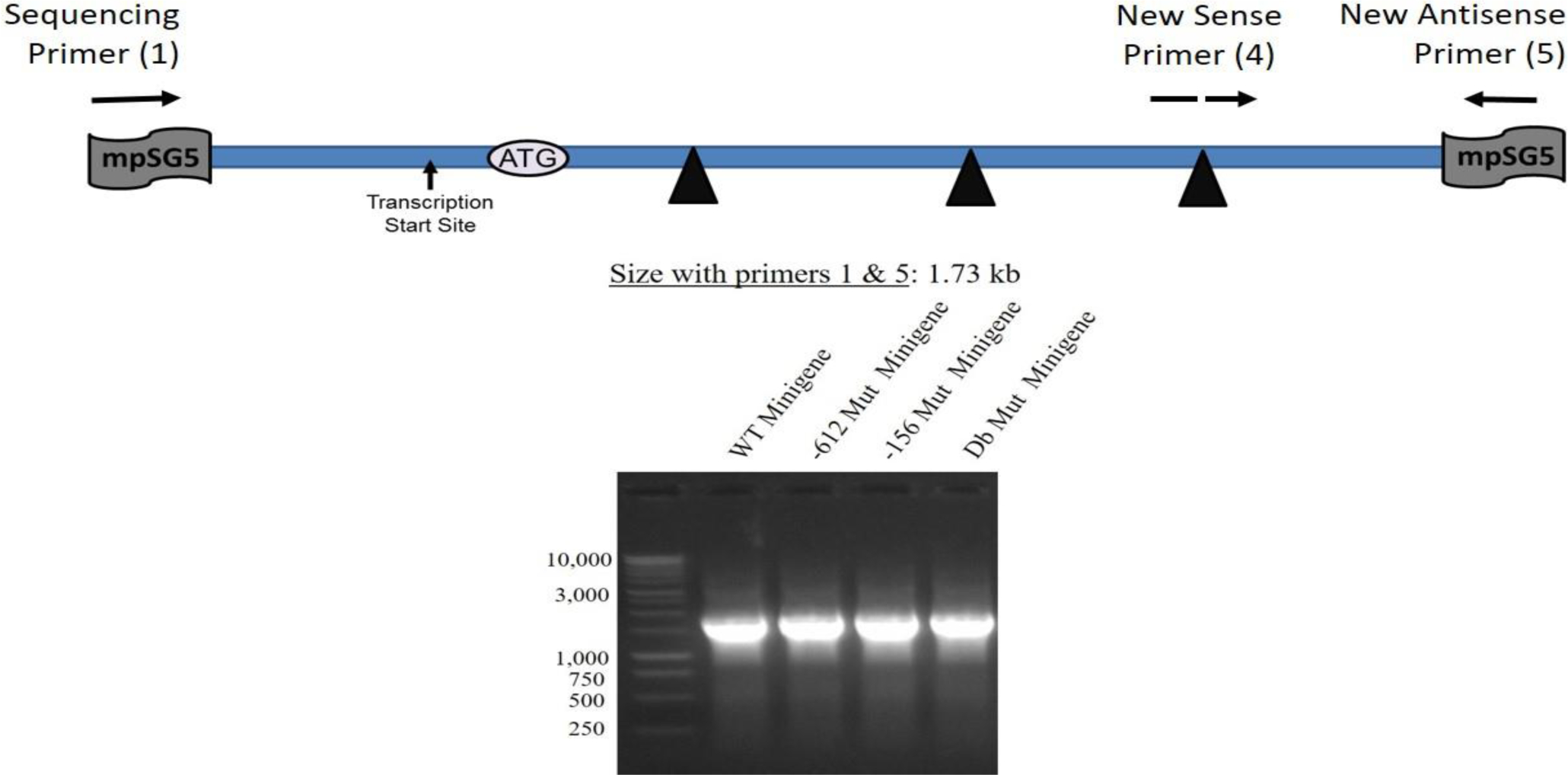
Testing of four minigene constructs using standard PCR. A schematic representation of the position of the primers used in the PCR relative to the minigene map, as well as the expected size of the amplified product, are shown. The same constructs (WT and three mutants) described in Fig. 7 were used here. A typical DNA electrophoresis of the PCR reactions is depicted.

**Figure 9:**
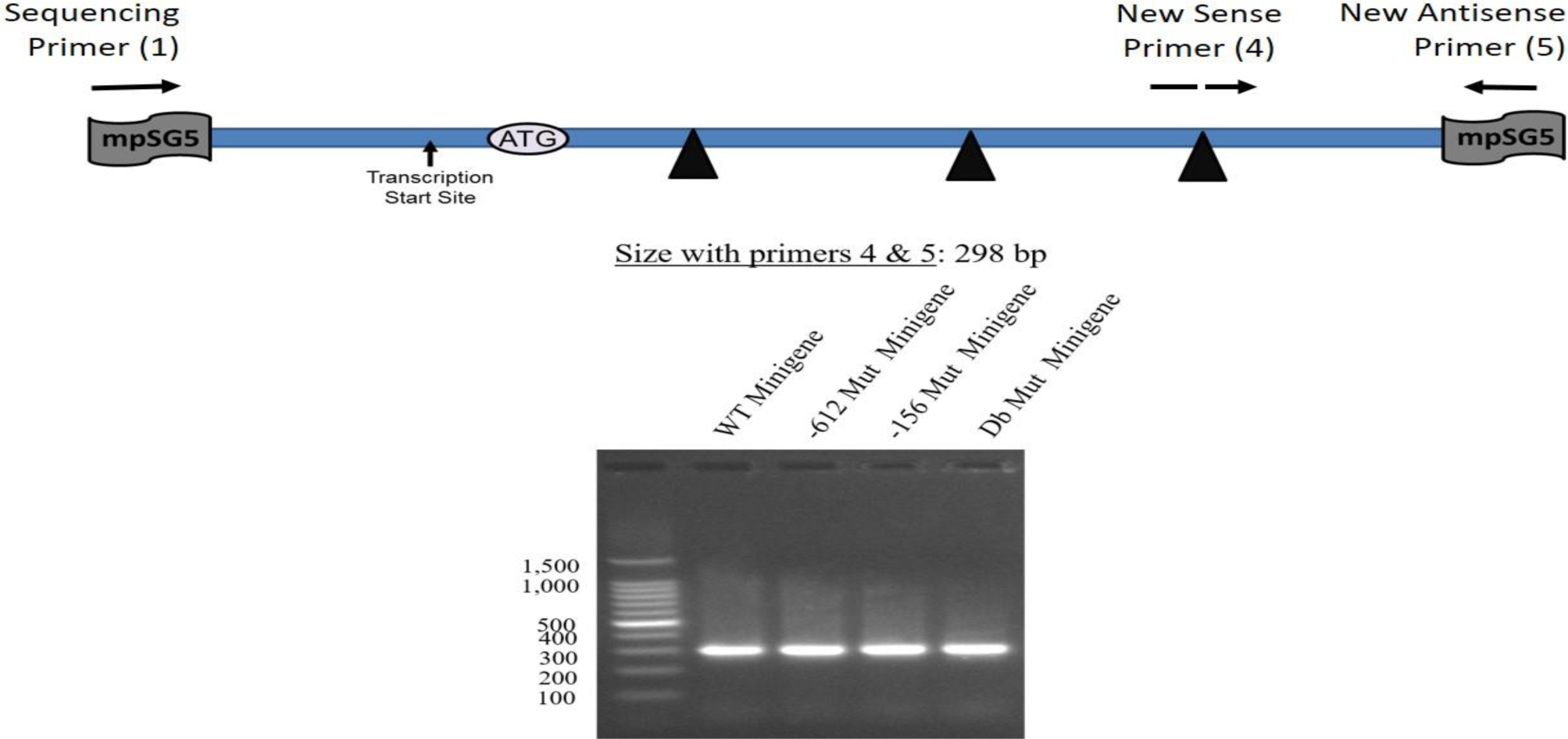
DNA electrophoresis illustrating the bands amplified by qRT-PCR using new sense primer (4) and new antisense primer (5) and the four minigene constructs described above. RNA and ssDNA preparation and analysis using qRT-PCR were done using the methods described in the text. An aliquot of the resulting reactions was used in the electrophoresis. A schematic representation of the position of the primers used in the qRT-PCR relative to the minigene map, as well as the expected size of the amplified product, are shown.

**Figure 10:**
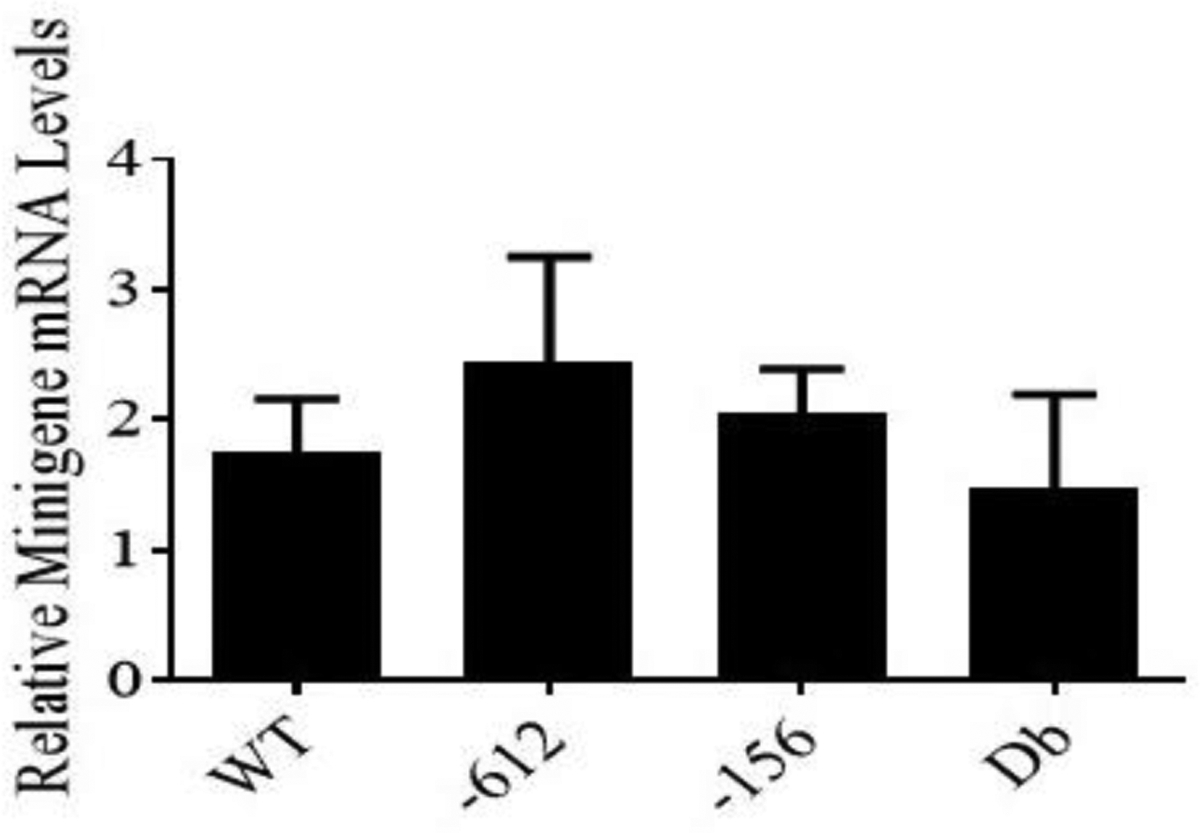
Calculations for the qRT-PCR described in Fig. 9 performed employing the comparative Ct method and the data obtained with primers specific for 18s rRNA. Representative data are shown for n=3 per construct.

**Figure 11:**
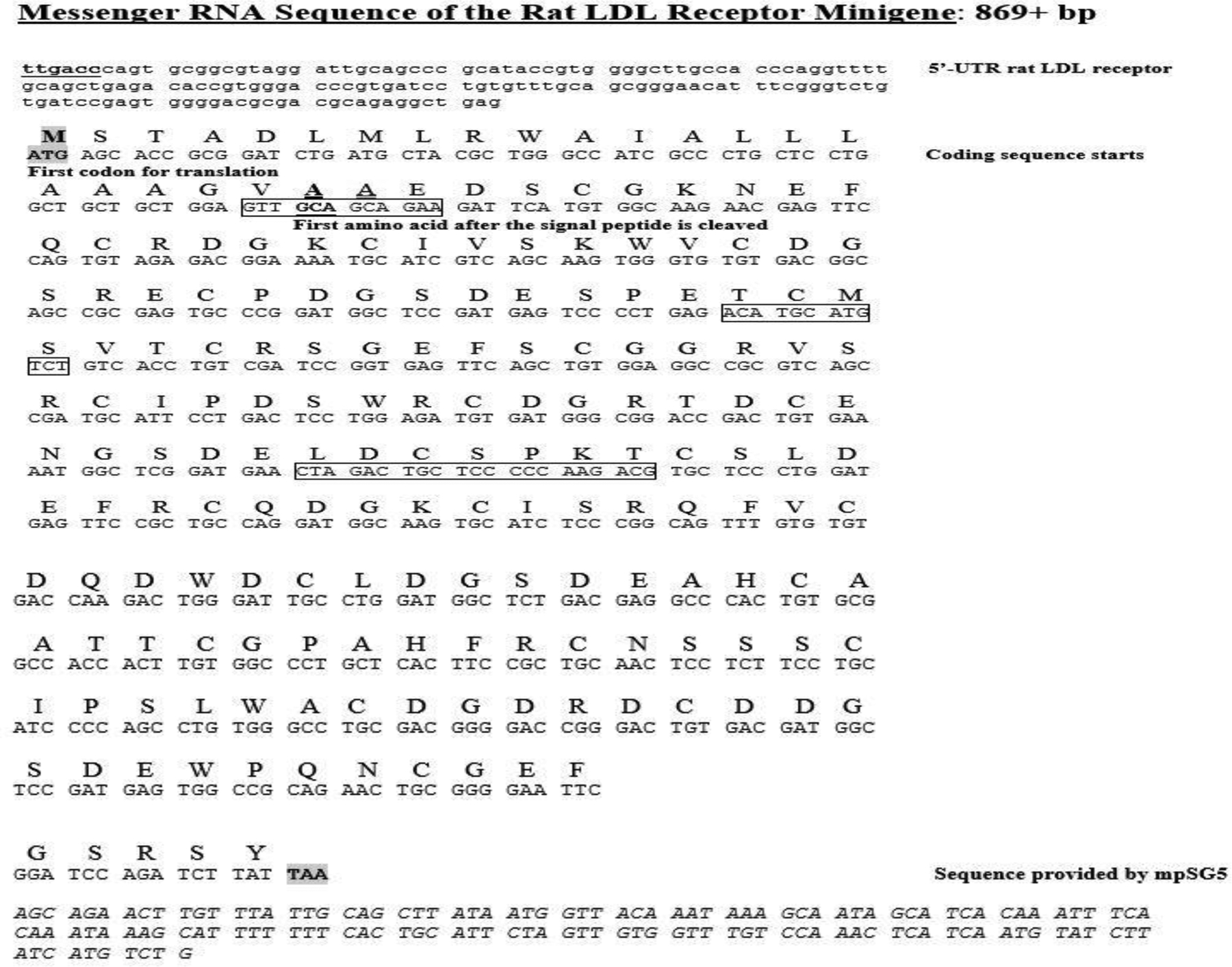
Predicted mRNA and protein sequences of the rat LDL receptor minigene construct. The first (ATG; provided by the minigene) and stop codons (provided by the mpSG5 vector) have been highlighted in grey. The first amino acid after the cleavage of the signal peptide for the truncated protein has been underlined. Rectangles indicate the exon junctions. The italic, uppercase sequence corresponds to the SV40 poly A region given by the mpSG5 vector.

Additional tests were performed on the four constructs including restriction enzyme analysis and standard PCR analysis. The results of the restriction enzyme analysis are shown in Figure 7. For this test, constructs were digested with Mlu I alone, Mlu I/Xho I, or Mlu I/EcoR I. As expected, no changes in the restriction enzyme map of these constructs occurred as a result of inserting the different mutations. Figure 8 illustrates that when standard PCR was performed using sequencing primer (1) and new antisense primer (5), the sizes of the PCR To test the functionality of the minigene constructs (WT and the three mutants), transfection studies were carried out in human hepatocyte-like C3A cells. RNA samples prepared from cells transfected with the different constructs were utilized in the synthesis of ssDNA for qRT-PCR analysis as described under Materials and Methods. The primers used in this experiment were new sense primer (4) and new antisense primer (5). After qRT-PCR, an aliquot of the PCR reaction was run on a DNA gel. Figure 9 shows that all the minigene constructs (WT and three mutants) spliced correctly since the amplified fragments had the expected size of about 300 bp. Since a single product was amplified in the qRT-PCR, quantitation of the results was performed using the comparative CT method as previously described [20]. As shown in Figure 10, there was no significant difference (p=0.703; n=3) in the basal levels of mRNA produced from the four constructs. Based on the analysis of the mRNA sequence produced from the WT minigene-mpSG5 construct (869+ bp; see Figure 11), it appears that this minigene encodes for an extracellular secreted protein of 18.7 kDa products produced from the four minigene constructs (WT and the three mutants) were the same. corresponding to the N-terminal region (not including the signal peptide) of the rat LDL receptor protein. Figure 11 illustrates the predicted mRNA and protein sequences of the rat LDL receptor minigene construct. The first codon (ATG; highlighted in grey) is provided by the minigene sequence. The first amino acid after cleavage of the signal peptide is underlined. The mpSG5 vector provides the stop codon (also highlighted in grey). Additional studies are required to confirm the production of the truncated LDL receptor protein produced by the minigene. This is the first minigene that has been reported for the LDL receptor of humans, mice, and rats. This minigene is not only functional by producing mRNA after splicing, but it also has the potential to secrete a truncated version of LDL receptor protein. This could allow adding fluorescent signals to the minigene-derived protein that could be used for quicker detection methods. The minigene could be used as a research tool to identify small molecules, natural products, and regulators of the LDL receptor gene that could be developed into LDL receptor-specific activators for therapeutic use.
